# Expiratory time constant for determinations of plateau pressure, respiratory system compliance, and total resistance

**DOI:** 10.1186/cc12500

**Published:** 2013-02-05

**Authors:** Nawar Al-Rawas, Michael J Banner, Neil R Euliano, Carl G Tams, Jeff Brown, A Daniel Martin, Andrea Gabrielli

**Affiliations:** 1 Department of Anesthesiology, College of Medicine, University of Florida, PO Box 100254, Gainesville, FL 32610-0254, USA; 2 Convergent Engineering, 107 SW 140th Terrace, #1, Newberry, FL 32669, USA; 3 Department of Physical Therapy, College of Public Health and Health Professions, University of Florida, PO Box 100154, Gainesville, FL 32610-0154, USA; 4 Department of Surgery, College of Medicine, University of Florida, PO Box 100254, Gainesville, FL, USA

## Abstract

**Introduction:**

We hypothesized the expiratory time constant (Ƭ_E_) may be used to provide real time determinations of inspiratory plateau pressure (Pplt), respiratory system compliance (Crs), and total resistance (respiratory system resistance plus series resistance of endotracheal tube) (Rtot) of patients with respiratory failure using various modes of ventilatory support.

**Methods:**

Adults (*n *= 92) with acute respiratory failure were categorized into four groups depending on the mode of ventilatory support ordered by attending physicians, i.e., volume controlled-continuous mandatory ventilation (VC-CMV), volume controlled-synchronized intermittent mandatory ventilation (VC-SIMV), volume control plus (VC+), and pressure support ventilation (PSV). Positive end expiratory pressure as ordered was combined with all aforementioned modes. Pplt, determined by the traditional end inspiratory pause (EIP) method, was combined in equations to determine Crs and Rtot. Following that, the Ƭ_E _method was employed, Ƭ_E _was estimated from point-by-point measurements of exhaled tidal volume and flow rate, it was then combined in equations to determine Pplt, Crs, and Rtot. Both methods were compared using regression analysis.

**Results:**

Ƭ_E_, ranging from mean values of 0.54 sec to 0.66 sec, was not significantly different among ventilatory modes. The Ƭ_E _method was an excellent predictor of Pplt, Crs, and Rtot for various ventilatory modes; r^2 ^values for the relationships of Ƭ_E _and EIP methods ranged from 0.94 to 0.99 for Pplt, 0.90 to 0.99 for Crs, and 0.88 to 0.94 for Rtot (*P *<0.001). Bias and precision values were negligible.

**Conclusions:**

We found the Ƭ_E _method was just as good as the EIP method for determining Pplt, Crs, and Rtot for various modes of ventilatory support for patients with acute respiratory failure. It is unclear if the Ƭ_E _method can be generalized to patients with chronic obstructive lung disease. Ƭ_E _is determined during passive deflation of the lungs without the need for changing the ventilatory mode and disrupting a patient's breathing. The Ƭ_E _method obviates the need to apply an EIP, allows for continuous and automatic surveillance of inspiratory Pplt so it can be maintained ≤ 30 cm H_2_O for lung protection and patient safety, and permits real time assessments of pulmonary mechanics.

## Introduction

Inspiratory plateau pressure (Pplt) or static elastic recoil pressure of the respiratory system is useful for assessing elastic and resistive properties of the respiratory system of patients with respiratory failure [1]. Pplt provides relevant diagnostic information and should be monitored routinely when applying ventilatory support because of the importance of maintaining it at ≤30 cm H_2_O for lung protection [2-4]. Increased Pplt is associated with increased respiratory system elastance or decreased respiratory system compliance (Crs) (lungs and chest wall), and vice versa. Increased differences between Pplt and peak airway pressure during inhalation (Paw) are associated with increased total inspiratory resistance (Rtot), which includes the series resistance of the endotracheal tube (ETT) plus physiologic airways resistance. Pplt is essential for determining Crs and Rtot as follows:

(1) $$\text{Crs = }\frac{\text{Tidal volume}}{\text{Pplt - Positive and expiratory pressure}}$$

(2) $$\text{Rtot = }\frac{\text{Paw - Pplt}}{\text{Inhaled flow rate}}$$

**Figure 1:**
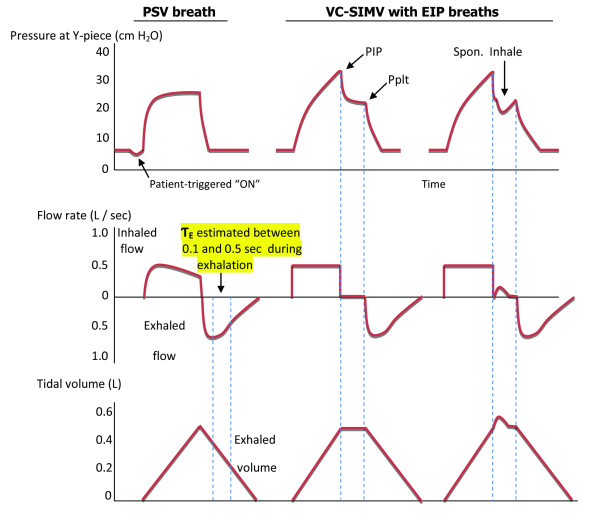
**Pressure, flow, and volume waveforms for determination of expiratory time constant**. In the first breath a patient is ventilated with pressure support ventilation (PSV) 20 cm H_2_O and positive end expiratory pressure (PEEP) 5 cm H_2_O. The expiratory time constant (Ƭ_E_) was estimated during passive exhalation between 0.10 and 0.50 seconds using exhaled flow rate and tidal volume waveform data (see Methods). In the second breath the same patient is temporarily changed to volume controlled synchronized intermittent mandatory ventilation (VC-SIMV) with an end inspiratory pause (EIP) for 0.5 second at PEEP 5 cm H_2_O, while applying similar peak inspiratory flow rate and tidal volume. This is done to determine peak airway pressure during inhalation (Paw) and inspiratory plateau pressure (Pplt) or static elastic recoil pressure of the respiratory system, needed for calculations of respiratory system compliance and total resistance (see equations 1 and 2). The third breath is an example of an EIP that is not tolerated, that is, the patient attempts to spontaneously inhale during the pause, precluding accurate measurement of Pplt.

Traditionally, Pplt is measured intermittently due to the need to temporarily modify ventilator settings, and to apply an end inspiratory pause (EIP) using volume-controlled ventilation. During an EIP, tidal volume (V_T_) is held within the lungs, and pressure at the airway opening decreases from Paw to Pplt. This assumes the patient is relaxed and complies with the EIP maneuver, as are those who are appropriately sedated and/or paralyzed, not breathing spontaneously, and receiving volume-controlled continuous mandatory ventilation (VC-CMV) (Figure 1). However, many patients receiving ventilatory support are not paralyzed, but are breathing spontaneously. For example, spontaneously breathing patients may receive pressure support ventilation (PSV) and/or volume-controlled synchronized intermittent mandatory ventilation (VC-SIMV). Temporarily applying an EIP maneuver during PSV may be problematic; it interferes with the patient's breathing, predisposing to patient and ventilator breathing asynchrony, that is, the patient attempts to spontaneously inhale and exhale during the pause, causing erroneous measurements of Pplt (Figure 1). In our experience this occurs approximately 75% of the time when an EIP is applied. Thus, accurate measurements of Pplt, and therefore determinations of Crs and Rtot, may at times be difficult to obtain.

An automatic and continuous method of determining Pplt, Crs and Rtot, that is not dependent on an EIP is desirable. We propose using the expiratory time constant (Ƭ_E_) for determinations of Pplt, Crs, and Rtot. Ƭ_E _contains information about the mechanical properties of the respiratory system, namely, elastance and resistance [5]. It is hypothesized that real-time determinations of inspiratory Pplt, Crs, and Rtot may be estimated from the passive deflation of the lungs by using Ƭ_E_, and combined with appropriate equations. Another purpose of the study was to demonstrate that the Ƭ_E _method can be used with various modes of ventilatory support.

## Materials and methods

In this Institutional Review Board (University of Florida, Gainesville Health Science Center Institutional Review Board, IRB-01)-approved study, 92 intubated adults with acute respiratory failure and compromised pulmonary mechanics from various causes were evaluated in a surgical ICU (Table 1). ETT sizes used were 7.0, 7.5, 8.0, and 8.5 mm internal diameter. Diagnoses included trauma (gunshot or stab wound, vehicle accident, traumatic brain injury), complex abdominal surgery (liver transplant, abdominal cancer, nephrectomy, abdominal aortic aneurysm), complex neurological surgery (cerebral aneurysm, subarachnoid hemorrhage, subdural hematoma, brain tumor resection), and medical complications and comorbidities (chronic obstructive pulmonary disease (COPD), sepsis, pancreatitis, congestive heart failure, pneumonia, renal failure, diabetes, colitis). Patients were categorized into four groups depending on the mode of ventilatory support ordered by the attending physician, that is, VC-CMV (*n *= 24), VC-SIMV (*n *= 13), volume control plus (VC+) (*n *= 32), and PSV (*n *= 23) (Table 1). VC+ breaths were applied as a mandatory pressure-controlled breath type for assist-control. VC+ modulates the applied airway pressure based on tidal volume feedback. Positive end expiratory pressure (PEEP) and fractional inhaled concentration of oxygen (FIO_2_), as ordered by attending physicians, was combined with all aforementioned modes. All groups had relatively equal percentages of patients with traumatic injuries, complex abdominal and neurological surgeries, and medical comorbidities. All were ventilated with the same type of ventilator (Puritan-Bennett, Pleasanton, CA, USA, Model 840). Because the study involved only respiratory monitoring of patients treated with routinely used modes of ventilatory support, a waiver of informed consent was granted. The majority of patients breathed spontaneously, and they were provided with analgesia as needed to maintain a Riker sedation-agitation scale (SAS) score of 4 [6]. Others were heavily sedated and/or paralyzed, provided with analgesia, and not breathing spontaneously (SAS score 1 to 2); these patients received VC-CMV. All patients were hemodynamically stable with mean arterial blood pressures between 70 and 88 mm Hg.

**Table 1:** Patient group data (total number of patients = 92)

	VC-CMV	VC-SIMV	VC+	PSV
Number	24	13*	32†	23
Age, years,	49 ± 19	52 ± 18	50 ± 20	53 ± 20
Weight, kg,	69 ± 30	68 ± 40	81 ± 30	78 ± 27
Gender, number	21 male 3 female	9 male* 4 female	19 male 13 female†	14 male 9 female
Breathing frequency, breaths/minute,	13 ± 8	32 ± 9†	16 ± 5	20 ± 6
V_T_, ml/kg IBW	8 ± 4	9 ± 4	6 ± 4	7 ± 4
Paw, cm H_2_O PEEP (cm H_2_O)	32 ± 6 7.7 ± 4.4	34 ± 7 9.9 ± 3.6	29 ± 8 7.9 ± 4	28 ± 8 7.9 ± 3
FIO_2_	0.43 ± 0.14	0.45 ± 0.10	0.45 ± 0.12	0.44 ± 0.09
Ƭ_E_, sec	0.58 ± 0.20	0.54 ± 0.27	0.66 ± 0.27	0.58 ± 0.18

Data presented as mean ± SD unless stated otherwise. For all patient groups pulse oximeter oxygen saturation ranged from 90% to 95% and partial pressure end-tidal carbon dioxide ranged from 30 to 41 mm Hg. *P *<0.05 for *lowest and ^†^greatest compared to other groups. VC-CMV, volume controlled-continuous mandatory ventilation; VC-SIMV, volume controlled-synchronized intermittent mandatory ventilation, VC+: volume control plus, PSV: pressure support ventilation, V_T_: tidal volume, IBW: Ideal body weight, Paw: peak airway pressure during inhalation, PEEP: positive end expiratory pressure, FIO_2_: fractional inhaled oxygen concentration, Ƭ_E_: expiratory time constant.

**Figure 2:**
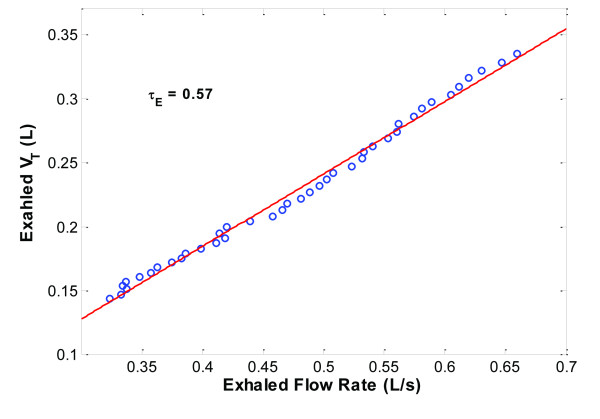
**Determination of expiratory time constant**. Example of determining the expiratory time constant (Ƭ_E_) for one patient in the study is shown. Ƭ_E _was determined by point-by-point analyses of exhaled tidal volume (V_T_) and flow rate values between 0.10 and 0.50 seconds, that is, the exhaled V_T _and flow rate curves are substituted by a fitted straight line (least squares fit) and the slope of the line is Ƭ_E_.

Ƭ_E _is expressed in units of time (seconds). One time constant represents the time required for the respiratory system to reach 63% of its equilibrium value and is an indication of the time required for the lungs to empty during exhalation [7,8]. Ƭ_E _was estimated from 0.10 to 0.50 seconds after the beginning of exhalation. Specifically, this was done using point-by-point measurements of exhaled volume (L) divided by corresponding point-by-point measurements of exhaled flow rate (L/sec) to generate a representation of exhalation; the linear slope of this representation (Figures 1 and 2) was Ƭ_E_. A straight-line (first-order model) relationship between exhaled volume and flow rate is important, because it indicates the subject has relaxed his/her respiratory muscles sufficiently to provide a reliable estimation of Ƭ_E _(Figure 2). If the relationship is not linear, the Ƭ_E _estimation is not valid and the procedure should be repeated [9]. The first part of exhalation (between 0.0 and 0.1 sec) was excluded for three reasons: (1) the time constant can only be determined for that part of exhalation that is exponential during lung emptying; the initial part of exhalation at peak exhaled flow rate cannot be analyzed using a first-order model as we and others used [9,10]; (2) to reduce possible interference from the ventilator's exhalation valve during initial opening, and (3) to reduce the interference of any residual patient breathing efforts. The end of exhalation was also excluded because of the resistance of the ventilator's PEEP/exhalation valve, which may become more significant at the end of exhalation and the possibility of late emptying lung compartment kinetic behavior, which may result in inaccurate determinations of Ƭ_E _because of alveolar emptying inequalities and time constant inhomogeneity within the lungs (Pendelluft effect) [11,12]. The value of Ƭ_E _obtained from this method reflects factors affecting lung emptying, namely, Crs, physiologic airways resistance, series resistance of ETT and PEEP/exhalation valve [10]. Resistance of the ETT was included in determinations of Ƭ_E _for all patients.

Although Ƭ_E _applies to the exhalation phase of breathing, it is a required mathematical term in equations for determining inspiratory Pplt, Crs, and Rtot. After Ƭ_E _was estimated, Pplt, Crs, and Rtot were determined using the following equations (derivations of equations are stated in Additional File 1):(3)(4)(5)

**Figure 3:**
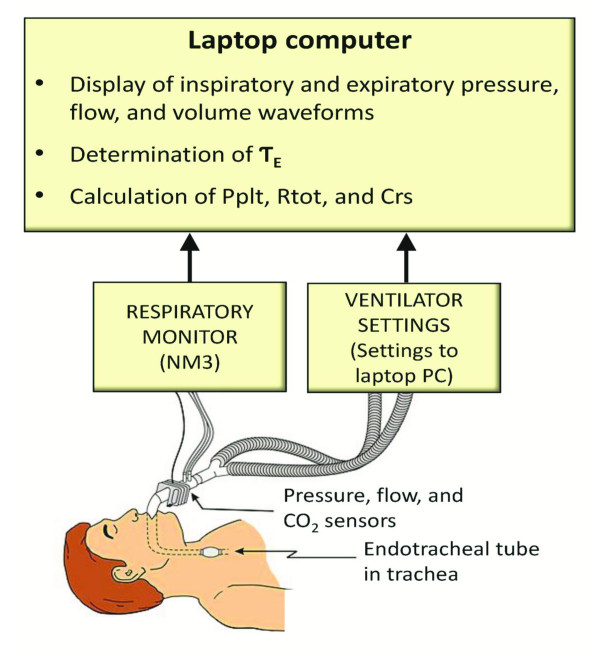
**Computerized bedside system for determining expiratory time constant and related parameters**. Data from pressure, flow, and carbon dioxide sensors, positioned between the endotracheal tube and ventilator breathing circuit, are directed to a monitor (NM3, Respironics) for measurements of pressure, flow, and volume. Data from the monitor are in turn directed to a laptop computer containing software (Convergent Engineering) for the determinations of expiratory time constant (Ƭ_E_), inspiratory plateau pressure (Pplt), total respiratory resistance (Rtot), and respiratory system compliance (Crs).

Routine monitoring of our ventilator-dependent patients includes applying an EIP at recommended four-hour intervals to monitor and maintain Pplt at ≤30 cm H_2_O[4],allowing for calculations of Crs and Rtot (Figure 1, equations 1 and 2). More specifically, the ventilator mode is switched to VC-SIMV using a V_T _of 6 ml/kg ideal body weight with an EIP time of 0.5 second. We recorded Pplt, Crs, and Rtot values using the EIP method. Immediately following this, Ƭ_E _was determined and then Pplt, Crs, and Rtot were determined as previously described (equations 3, 4, and 5). Pplt, Crs, and Rtot values obtained from the EIP method were considered reference values; Pplt, Crs, and Rtot values obtained from the Ƭ_E _method were compared to the reference values. Data from a combined pressure, flow, and carbon dioxide sensor, positioned between the ETT and Y-piece of the ventilator breathing circuit, were directed to a respiratory monitor (NM3, Respironics, Hartford, CN, USA) and laptop computer with software (Convergent Engineering, Gainesville, FL, USA) to record airway pressure, flow rate, and V_T _waveforms and for determinations of Ƭ_E_, Pplt, Crs, and Rtot using the EIP and Ƭ_E _methods (Figure 3).

It was necessary to apply multiple EIP breaths to obtain an appropriate Pplt for some patients breathing spontaneously, for example, those receiving PSV. Many of these patients attempted to inhale and exhale during the pause (Figure 1, third breath), these breaths were not used. Those EIP breaths where patients did not inhale and exhale during the pause and in whom airway pressure decreased passively, generating a smooth plateau, (Figure 1, second breath) were used for determinations of Pplt. On average, EIP breaths were applied 5 to 10 times over a 15-minute period. A computer software code (Matlab) was developed to automatically verify valid pressure plateau segments. The software was programmed to find segments of the EIP with the following criteria: maximum tidal volume value, zero flow, and a horizontal or flat pressure plateau for a length equal to approximately 0.5 seconds. Manual visual inspection of every patient's pressure, flow, and volume waveforms were performed to verify the values garnered by the software.

Data were analyzed using regression analyses to evaluate relationships of the Ƭ_E _method for determining Pplt, Crs, and Rtot with the EIP method for determining Pplt, Crs, and Rtot, using analysis of variance (ANOVA), Fisher's exact test, and Bland and Altman analyses [13]. Data are mean ± SD; alpha was set at 0.05 for statistical significance.

## Results

There were no significant differences in Ƭ_E _among groups. Ƭ_E _was 0.58 ± 0.20 sec for the VC-CMV group, 0.54 ± 0.27 sec for the VC-SIMV group, 0.66 ± 0.27 sec for the VC+ group, and 0.58 ± 0.18 sec for the PSV group.

Pplt values ranged from 11 to 38 cm H_2_O, 13 to 38 cm H_2_O, 11 to 38 cm H_2_O, and 12 to 33 cm H_2_O for the VC-CMV, VC-SIMV, VC+, and PSV groups, respectively. The r^2 ^values for the relationships of Ƭ_E _and EIP methods were 0.99, 0.99, 0.98, and 0.94 (*P *<0.001) for the VC-CMV, VC-SIMV, VC+, and PSV groups, respectively.

Crs values ranged from 0.02 to 0.095 L/cm H_2_O, 0.015 to 0.09 L/cm H_2_O, 0.022 to 0.092 L/cm H_2_O, and 0.022 to 0.068 L/cm H_2_O for the VC-CMV, VC-SIMV, VC+, and PSV groups, respectively. The r^2 ^values for the relationships of Ƭ_E _and EIP methods were 0.99, 0.98, 0.97, and 0.90 (*P *<0.001) for VC-CMV, VC-SIMV, VC+, and PSV groups, respectively.

Rtot values ranged from 8 to 21 cm H_2_O/L/sec, 8 to 15.5 cm H_2_O/L/sec, 5 to 23 cm H_2_O/L/sec, and 5 to 17 cm H_2_O/L/sec for the VC-CMV, VC-SIMV, VC+, and PSV groups respectively. The r^2 ^values for the relationships of the Ƭ_E _and EIP methods were 0.92, 0.94, 0.88, and 0.91 (*P *<0.001) for the VC-CMV, VC-SIMV, VC+, and PSV groups, respectively.

**Figure 4:**
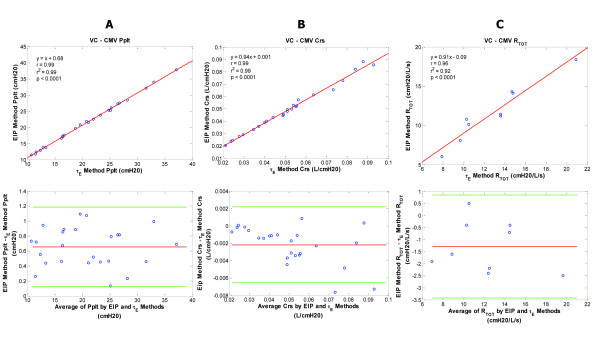
**Plateau pressure, compliance, and resistance data for the volume controlled-continuous mandatory ventilation (VC-CMV) patient group**. VC-CMV patient group - regression and corresponding Bland-Altman plots for inspiratory plateau pressure (Pplt) (**A**), respiratory system compliance (Crs) (**B**), and total respiratory resistance (Rtot) (**C**) are shown comparing the end inspiratory pause (EIP) and expiratory time constant (Ƭ_E_) methods. Pplt bias = 0.66; Pplt precision = 0.40 to 0.93; Pplt limits of agreement = 0.13 to 1.18. Crs bias = -0.002; Crs precision = -0.005 to 0.000; Crs limits of agreement = -0.007 to 0.002. Rtot bias = -1.29; Rtot precision = -2.36 to -0.22; Rtot limits of agreement = -3.43 to 0.85.

**Figure 5:**
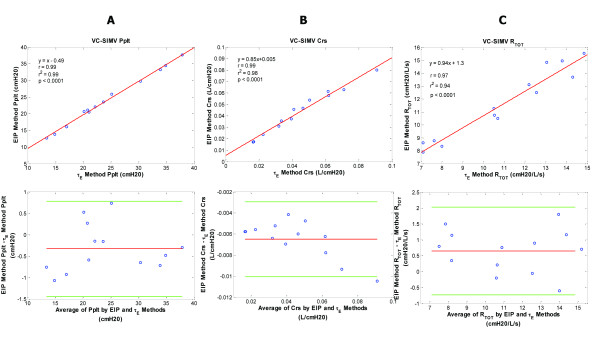
**Plateau pressure, compliance, and resistance data for the volume controlled-synchronized intermittent mandatory ventilation (VC-SIMV) patient group**. VC-SIMV patient group - regression and corresponding Bland-Altman plots for inspiratory plateau pressure (Pplt) (**A**), respiratory system compliance (Crs) (**B**), and total respiratory resistance (Rtot) (**C**) are shown comparing the end inspiratory pause (EIP) and expiratory time constant methods (Ƭ_E_). Pplt bias = -0.33; Pplt precision = -0.89 to 0.23; Pplt limits of agreement = -1.44 to 0.78. Crs bias = -0.0065; Crs precision = -0.005 to -0.008; Crs limits of agreement = -0.01 to -0.003. Rtot bias = 0.65; Rtot precision = -0.04 to 1.34; Rtot limits of agreement = -0.73 to 2.03.

**Figure 6:**
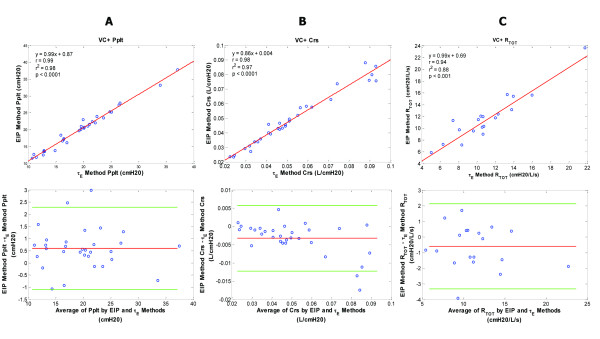
**Plateau pressure, compliance and resistance data for the volume control plus (VC+) patient group**. VC+ patient group - regression and corresponding Bland-Altman plots for inspiratory plateau pressure (Pplt) (**A**), respiratory system compliance (Crs) (**B**), and total respiratory resistance (Rtot) (**C**) are shown comparing the end inspiratory pause (EIP) and expiratory time constant (Ƭ_E_) methods. Pplt bias = 0.6; Pplt precision = -0.25 to 1.44; Pplt limits of agreement = -1.09 to 2.28. Crs bias = -0.003; Crs precision = -0.008 to 0.001; Crs limits of agreement = -0.012 to 0.006. Rtot bias = -0.6; Rtot precision = -1.96 to 0.77; Rtot limits of agreement = -3.32 to 2.13.

**Figure 7:**
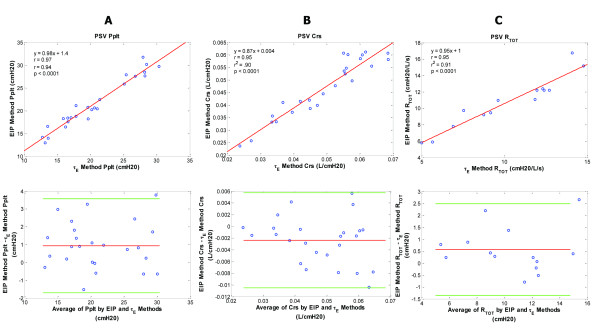
**Plateau pressure, compliance, and resistance data for the pressure support ventilation (PSV) patient group**. PSV patient group - regression and corresponding Bland-Altman plots for inspiratory plateau pressure (Pplt) (**A**), respiratory system compliance (Crs) (**B**), and total respiratory resistance (Rtot) (**C**) are shown comparing the end inspiratory pause (EIP) and expiratory time constant (Ƭ_E_) methods. Pplt bias = 0.94; Pplt precision = -0.38 to 2.26; Pplt limits of agreement = -1.70 to 3.58. Crs bias = -0.002; Crs precision = -0.006 to 0.002; Crs limits of agreement = -0.011 to 0.006. Rtot bias = 0.57; Rtot precision = -0.39 to 1.53; Rtot limits of agreement = -1.35 to 2.49.

Bias and precision values for all correlations and for all ventilatory groups were minimal (Figures 4, 5, 6, and 7). More patients were in the VC+ group than other groups. This may be due to chance occurrence; VC+ was a more commonly used mode of ventilatory support at the time of the study. Also, there were gender differences among groups. Breathing frequency was highest in the VC-SIMV group (Table 1). These issues did not influence the pressure, flow, and volume waveform data used for the EIP and Ƭ_E _methods. There were no significant differences in age, weight, V_T_, Peak inflation pressure (PIP), PEEP, and FIO_2 _among groups.

## Discussion

The Ƭ_E _method provided appropriate determinations and was an excellent predictor of Pplt, Crs, and Rtot for patients with acute respiratory failure receiving various modes of ventilatory support. It predicted or explained 94 to 99% of the variance in determining Pplt, 90 to 99% of the variance in determining Crs, and 88 to 94% of the variance in determining Rtot for patients receiving VC-CMV, VC-SIMV, VC+, and PSV. Bias and precision values were negligible for all measurements.

Crs ranged from near normal values at 0.095 L/cm H_2_0 to much lower values at 0.022 L/cm H_2_O, indicative of patients with stiff non-compliant lungs. Rtot for some patients was higher than normal and in the range of 15 to 23 cm H_2_O/L/sec, although most did not have COPD. We speculate that the increased Rtot values may have been due to increased physiologic airways resistance and/or increased imposed resistance of the ETT as a result of internal narrowing of the tube due to secretions. The values for Crs and Rtot obtained in this study span ranges from moderate to more compromised forms of impaired pulmonary mechanics of patients with respiratory failure.

For healthy, non-intubated adults, Ƭ_E _was reported in the range of 0.38 to 0.51 seconds [9]. The mean values of Ƭ_E _determined in this study for patients with acute respiratory failure were larger and comparable to other studies of non-COPD patients with acute respiratory failure at similar levels of PEEP. Guttmann *et al. *[10] reported an average Ƭ_E _of 0.60 seconds and Kondili *et al. *[14] reported Ƭ_E _to be in the range of 0.70 seconds. In another study of COPD patients, Ƭ_E _varied inversely with PEEP; at end exhalation Ƭ_E _ranged up to 3.75 seconds on zero PEEP and up to 1.58 seconds on 10 cm H_2_0 PEEP [15].

A potential limitation of our study is that only two patients had COPD. For COPD patients with increased airways resistance and Crs, Ƭ_E _may be longer than in our patients with acute respiratory failure. This does not imply the Ƭ_E _method for determining Pplt, Crs, and Rtot cannot be employed for COPD patients; we speculate it may be effective for these patients. It is unclear if our method can be generalized to patients with COPD with abnormally long expiratory time constants. A follow up study of COPD patients would offer additional insight.

In normal, non-intubated adults, McIlroy *et al*. initially described using exhaled V_T _and flow rate to construct a line, and the slope of the line reflected Ƭ_E _[9]. This was done using an X-Y plotter and a complicated process of determining angular tangents of the slope. Using a test lung and dogs, Brunner *et al*. modified this method by applying multiple correction factors, equations, and mathematical modeling to determine Ƭ_E _[16]. In adults with acute respiratory failure, Guttmann *et al*. discarded the initial portion of the exhaled flow rate tracing just after the inflection point (point of greatest flow rate at the onset of exhalation) and then divided the exhaled V_T _curve into five slices to determine representative expiratory time constant components for each slice [10]. The slope of straight lines fitted to the exhaled V_T _and flow rate curve within each slice was then used to determine an average value for Ƭ_E_. The aforementioned methods were done by hand, and were unwieldy, complicated, and time-consuming processes. They are impractical for clinical use, in contrast to our method. Our approach simplifies these methods by using only the 0.10 to 0.50 seconds portion of the exhaled V_T _and flow rate curves to derive a slope for determination of Ƭ_E_, and then use Ƭ_E _in equations for determinations of Pplt, Crs, and Rtot. Additionally, the approach is automated by using the rapid processing speed of a laptop computer with appropriate software at the bedside to generate real time determinations of Pplt, Crs, and Rtot; a practical easy-to-use clinical method (Figure 3).

Ƭ_E _as determined in this study may be considered as the total expiratory time constant, it includes physiologic and breathing apparatus components. Crs and bronchial airways resistance reflect the physiologic expiratory time constant component, and the series flow resistance of the ETT and PEEP/exhalation breathing valve constitute the breathing apparatus expiratory time constant component. The expiratory time constants for these various components were quantified in intubated adults with acute respiratory failure (non-COPD patients) [10]. The physiologic expiratory time constant was lowest at 0.30 seconds (average), the physiologic expiratory time constant plus flow resistive time constant of the ETT was higher at 0.50 seconds (average), and the physiologic expiratory time constant, plus the combined flow resistive time constants of the ETT, ventilator circuit, and PEEP/exhalation valve, that is, the total Ƭ_E_, was highest at 0.65 seconds, similar to values we determined (Table 1). It was not a purpose of our study to differentiate and quantify physiologic and expiratory time constant components for intubated patients with respiratory failure connected to a ventilator. A purpose was to demonstrate an automatic method for determining the total expiratory time constant, which is of practical concern because it reflects the rate of lung emptying for intubated patients receiving ventilatory support, and because Ƭ_E _can be used for determinations of Pplt, Crs, and Rtot.

Another purpose of the study was to demonstrate that the Ƭ_E _method for determining Pplt, Crs, and Rtot was valid for representative forms of ventilatory support. Figures 4, 5, 6 and 7 illustrate comparable values for Pplt, Crs, and Rtot using the Ƭ_E _and traditional EIP methods for various ventilatory modes. To add additional patient groups receiving other forms of positive pressure ventilation, we believe is unnecessary because the Ƭ_E _method is predicated on passive deflation of the lungs using exhaled tidal volume and flow waveforms.

The Ƭ_E _method obviates the need to temporarily change modes and apply a VC-SIMV breath with an EIP. At times, the EIP method may be impractical. Consider a spontaneously breathing patient treated with PSV and PEEP, for example, in whom applying an EIP is uncomfortable and disrupts the breathing pattern. During the pause, the patient may not remain passive and attempt to breathe, precluding accurate estimates of Pplt, and thus, Crs and Rtot. As a result, the clinician may become frustrated and forego continued attempts to apply an EIP. Consequently, vital information about the patient's pulmonary elastance and resistance is denied. It is important to assess and follow changes in pulmonary mechanics due to effects of disease and/or therapeutic maneuvers applied to the lungs. For example, the before and after effects of PEEP on Crs, as well as the before and after effects of bronchodilator therapy on Rtot can be assessed.

A patient safety/lung protection implication involves continuous surveillance of Pplt by using the Ƭ_E _method. Lung protective strategies for patients with acute lung injury call for limiting Pplt to ≤30 cm H_2_O and preventing lung stretch to protect the lungs from physical trauma to lung tissue [2,3]. Increased Pplt associated with V_T _should be avoided; it is associated with ventilator-induced lung injury [17,18]. Because real-time Pplt values are generated using the Ƭ_E _method, if Pplt acutely increased to 45 cm H_2_O, for example, the clinician would be alerted to this potentially injurious pressure and intervene to lower Pplt (open-loop approach). Contrast the Ƭ_E _method to the current traditional practice of applying an EIP once every four or eight hours, for example, where acute increases in Pplt may go undetected for long periods. Pplt values >30 cm H_2_0 occurred at times for some patients in our study. When made aware of these pressures using the traditional EIP method, V_T _was decreased in 1-ml/kg steps (minimal V_T _4 ml/kg) [2,4] to maintain Pplt at ≤30 cm H_2_O. Had Pplt been monitored continuously using the Ƭ_E _method, Pplt >30 cm H_2_O for long periods could have been avoided. If a ventilator's operating software employed the Ƭ_E _method for determining Pplt, then as Pplt increased to inappropriately high levels, the ventilator would immediately alert the clinician and automatically intervene to limit Pplt to ≤30 cm H_2_O by decreasing V_T _as stated above (closed-loop approach).

## Conclusions

In conclusion, Pplt, Crs, and Rtot may be derived automatically and continuously by using Ƭ_E _from passive deflation of the lungs for various modes of ventilatory support. The Ƭ_E _method was just as good as the traditional EIP method for determining Pplt, Crs, and Rtot for patients with acute respiratory failure. The Ƭ_E _method obviates the need to apply a volume-controlled breath with an EIP, which may be impractical for many intubated, spontaneously breathing patients. Real-time monitoring of pulmonary mechanics during ventilatory support are facilitated using the Ƭ_E _method.

## Key messages

• The expiratory time constant (Ƭ_E_) may be determined in real-time for patients receiving ventilatory support using point-by-point analyses of exhaled tidal volume and flow waveform data.

• Ƭ_E _is combined in equations allowing for real-time determinations of Pplt, Crs, and Rtot (respiratory system resistance, plus series resistance of endotracheal tube and ventilator breathing apparatus) for ventilator-dependent patients.

• The Ƭ_E _method for determining Pplt, Crs, and Rtot was compared with the traditional EIP method for determining Pplt, Crs, and Rtot for four forms of ventilatory support, namely, volume controlled-continuous mandatory ventilation, volume controlled-synchronized intermittent mandatory ventilation, pressure control plus, and pressure support ventilation. The r^2 ^values for the relationships of Ƭ_E _and EIP methods ranged from 0.94 to 0.99 for Pplt, 0.90 to 0.99 for Crs, and 0.88 to 0.94 for Rtot (*P *<0.001). Bias and precision values were negligible.

• The Ƭ_E _method obviates the need to disrupt the breathing pattern with an EIP, a requirement for determination of Pplt, Crs, and Rtot.

• For patient safety/lung protection, continuous surveillance of Pplt is achieved using the Ƭ_E _method. Lung protective strategies for patients with acute lung injury call for limiting Pplt to ≤30 cm H_2_O and preventing lung stretch to protect the lungs from physical trauma to lung tissue.

## Abbreviations

ANOVA: analysis of variance; COPD: chronic obstructive pulmonary disease; Crs: respiratory system compliance; EIP: end inspiratory pause; ETT: endotracheal tube; FIO_2_: fractional inhaled oxygen concentration; IRB: internal review board; Paw: peak airway pressure during inhalation; PEEP: positive end expiratory pressure; PetCO_2_: partial pressure end-tidal carbon dioxide; PIP: peak inflation pressure; Pplt: inspiratory plateau pressure; PSV: pressure support ventilation; Rtot: total resistance; SAS score: Riker sedation-agitation scale score; SpO_2_: pulse oximeter oxygen saturation; Ƭ_E_: expiratory time constant; VC+: volume control plus; VC-CMV: volume controlled-continuous mandatory ventilation; VC-SIMV: volume controlled-synchronized intermittent mandatory ventilation; V_T_: tidal volume.

## Competing interests

Dr. Banner is a consultant for Convergent Engineering. Dr. Euliano is President of Convergent Engineering and Dr. Tams is an associate of Convergent Engineering (developer of software used in study). All other authors declare that they have no competing interests. The authors received clinical research funds from Respironics Inc., the study sponsor. Dr. Euliano holds stocks in Convergent Engineering. The authors have applied for a patent.

## Authors' contributions

MJB co-developed ideas for the study, directed clinical data collection, analyzed all data, and wrote all manuscript drafts, conceived all figures, and reviewed literature for reference material. NA-R conceived the initial concept for the study, derived equations for the concept, did all initial calculations of data to prove correctness of concept, assisted in identifying patients to be studied, collected clinical data, and contributed to preparation of the manuscript. NRE co-developed ideas for study, developed software used in the study, performed statistical analyses, co-derived equations used in the study, and contributed to preparation of the manuscript. CGT performed statistical analyses and contributed to preparation of the manuscript. JB co-developed ideas for the study, assisted in identifying patients to be studied, and collected clinical data. ADM co-developed ideas for the study and contributed to preparation of the manuscript. AG co-developed ideas for the study, identified patients to be studied, directed clinical care aspects of the study, and contributed to preparation of the manuscript. All authors have read and approved the manuscript for publication.

## Authors' information

Sources of funding: Dr. Banner: State of Florida and Convergent Engineering; Dr. Al-Rawas: University of Florida, College of Medicine; Dr. Euliano: Convergent Engineering; Dr. Tams: Convergent Engineering; Mr. Brown: Shands Hospital at the University of Florida; Dr. Gabrielli: University of Florida, College of Medicine and Convergent Engineering.

## Supplementary Material

Additional file 1 **Derivations of equations**. Derivations of equations using the expiratory time constant (Ƭ_E_) method. Click here for file
