# Supplementary material for: Expiratory time constant for determinations of plateau pressure, respiratory system compliance, and total resistance
Source: Crit Care. 2013 Feb 5;17(1):R23. doi: 10.1186/cc12500 (PMC4056774; doi:10.1186/cc12500)
Supplement: Additional file 1 — Derivations of equations. Derivations of equations using the expiratory time constant (ƬE) method. [file cc12500-S1.DOCX]

**Additional File**

**Derivations of respiratory system compliance (Crs), total resistance (Rtot), and plateau pressure (Pplt) equations using the expiratory time constant (Ƭ_E_) method**

Traditionally, the time constant of the respiratory system for an intubated patient is the product of the Crs and Rtot. During inhalation, Rtot includes the series resistance of the endotracheal tube plus physiologic airways resistance. Crs, Rtot, and Pplt values determined apply to the inspiratory phase of breathing. Accordingly, inhaled flow is applicable to the following equations.

As described in the literature, the Ƭ_E_ can be estimated in real-time by dividing exhaled volume by exhaled flow during passive exhalation, i. e., Ƭ_E_ (t) = Exhaled volume (t) / Exhaled flow (t). Although associated with the exhalation phase of breathing, Ƭ_E_ is a required mathematical term in the following equations for determining Crs, Rtot, and Pplt.

After determining Ƭ_E_ (see Methods section), Crs can be derived as follows:

Paw – PEEP = V_T_ / Crs + Rtot X Inhaled flow

Where Paw is peak pressure during inhalation and PEEP is positive end expiratory pressure. By multiplying both sides of the equation by Crs, yields:

Crs (Paw – PEEP) = V_T_ + Rtot X Crs X Inhaled flow

Substituting Ƭ_E_  for Rtot X Crs on the right side yields:

Crs (Paw – PEEP) = V_T_ + Ƭ_E_  X Inhaled flow

Dividing by (Paw – PEEP) gives:

***V_T_ + Ƭ_E_ X Inhaled flow***

***Crs =***

***Paw - PEEP***

Similarly, for Rtot, start with the equation:

Paw – PEEP = V_T_ / Crs + Rtot X Inhaled flow

Multiply the term V_T_ / Crs by Rtot / Rtot gives:

Paw – PEEP = V_T_  X Rtot / Crs X Rtot + Rtot X Inhaled flow

Substituting Ƭ_E_  for Rtot X Crs on the right side gives:

Paw – PEEP = V_T_  X Rtot / Ƭ_E_  + Rtot X Inhaled flow

Simplifying the right side gives:

Paw – PEEP = R_RS_ (V_T_ / Ƭ_E_  + Inhaled flow)

Dividing both sides by ( V_T_ / Ƭ_E_  + Inhaled flow) gives:

***Paw - PEEP***

***Rtot =***

***V_T_***

***+ Inhaled flow***

***Ƭ_E_***

Lastly, for Pplt, use Pplt = (V_T_ / Crs) + PEEP to provide:

***(V_T_ X Paw) – (V_T_ X PEEP)***

***Pplt = PEEP +***

***V_T_ + Ƭ_E_ X Inhaled flow***
